# HMGB1/RAGE axis in tumor development: unraveling its significance

**DOI:** 10.3389/fonc.2024.1336191

**Published:** 2024-03-01

**Authors:** Anqi Fan, Mengxiang Gao, Xuhuan Tang, Mengya Jiao, Chenchen Wang, Yingying Wei, Quan Gong, Jixin Zhong

**Affiliations:** ^1^ College of Life Science, Yangtze University, Jingzhou, Hubei, China; ^2^ Department of Immunology, School of Basic Medicine, Tongji Medical College and State Key Laboratory for Diagnosis and Treatment of Severe Zoonostic Infectious Disease, Huazhong University of Science and Technology, Wuhan, Hubei, China; ^3^ National Demonstration Center for Experimental Basic Medical Education, School of Basic Medicine, Tongji Medical College, Huazhong University of Science and Technology, Wuhan, China; ^4^ Department of Rheumatology and Immunology, Tongji Hospital, Huazhong University of Science and Technology, Wuhan, Hubei, China; ^5^ Department of Immunology, School of Medicine, Yangtze University, Jingzhou, Hubei, China

**Keywords:** HMGB1, RAGE, HMGB1/RAGE axis, tumor, development

## Abstract

High mobility group protein 1 (HMGB1) plays a complex role in tumor biology. When released into the extracellular space, it binds to the receptor for advanced glycation end products (RAGE) located on the cell membrane, playing an important role in tumor development by regulating a number of biological processes and signal pathways. In this review, we outline the multifaceted functions of the HMGB1/RAGE axis, which encompasses tumor cell proliferation, apoptosis, autophagy, metastasis, and angiogenesis. This axis is instrumental in tumor progression, promoting tumor cell proliferation, autophagy, metastasis, and angiogenesis while inhibiting apoptosis, through pivotal signaling pathways, including MAPK, NF-κB, PI3K/AKT, ERK, and STAT3. Notably, small molecules, such as miRNA-218, ethyl pyruvate (EP), and glycyrrhizin exhibit the ability to inhibit the HMGB1/RAGE axis, restraining tumor development. Therefore, a deeper understanding of the mechanisms of the HMGB1/RAGE axis in tumors is of great importance, and the development of inhibitors targeting this axis warrants further exploration.

## 1 Introduction

Tumors are abnormal growths that occur when a specific cell within the body loses its normal growth regulation at the genetic level due to various tumorigenic factors. Consequently, tumor cells exhibit abnormal morphology, metabolism, and function, often losing their ability to differentiate and mature properly. Tumors are a major health concern, and despite the current treatment options, including surgery, radiation therapy, and chemotherapy, the outcomes remain unsatisfactory in terms of their effectiveness. However, in recent years, novel treatment modalities such as targeted therapy and immunotherapy have emerged, largely due to our advanced understanding of the molecular mechanisms of tumor development.

The High Mobility Group Protein 1 (HMGB1), which is categorized as a damage-associated molecular pattern (DAMP) molecule, is widely expressed in the nuclei of eukaryotic cells. Under normal circumstances, it remains in the nucleus. However, it can be actively secreted or passively released into the extracellular space upon stimulation or under stressed conditions. Due to its various locations and interactions with different receptors, HMGB1 exhibits a multitude of functions. Notably, when released into the extracellular space, its interaction with its receptors, such as the receptor for advanced glycation end products (RAGE), influences downstream signaling pathways, further contributing to its impact on various cellular functions. Recent studies have revealed that the HMGB1/RAGE axis is intricately involved in cell proliferation, apoptosis, metastasis, autophagy, and angiogenesis in various types of malignant tumors. In this review, provide an overview of the diverse functions of the HMGB1/RAGE axis in tumor development and delve into the associated signaling pathways, aiming to illustrate its potential role in tumorigenesis and therapeutic intervention.

### 1.1 HMGB family proteins

**Figure 1:**
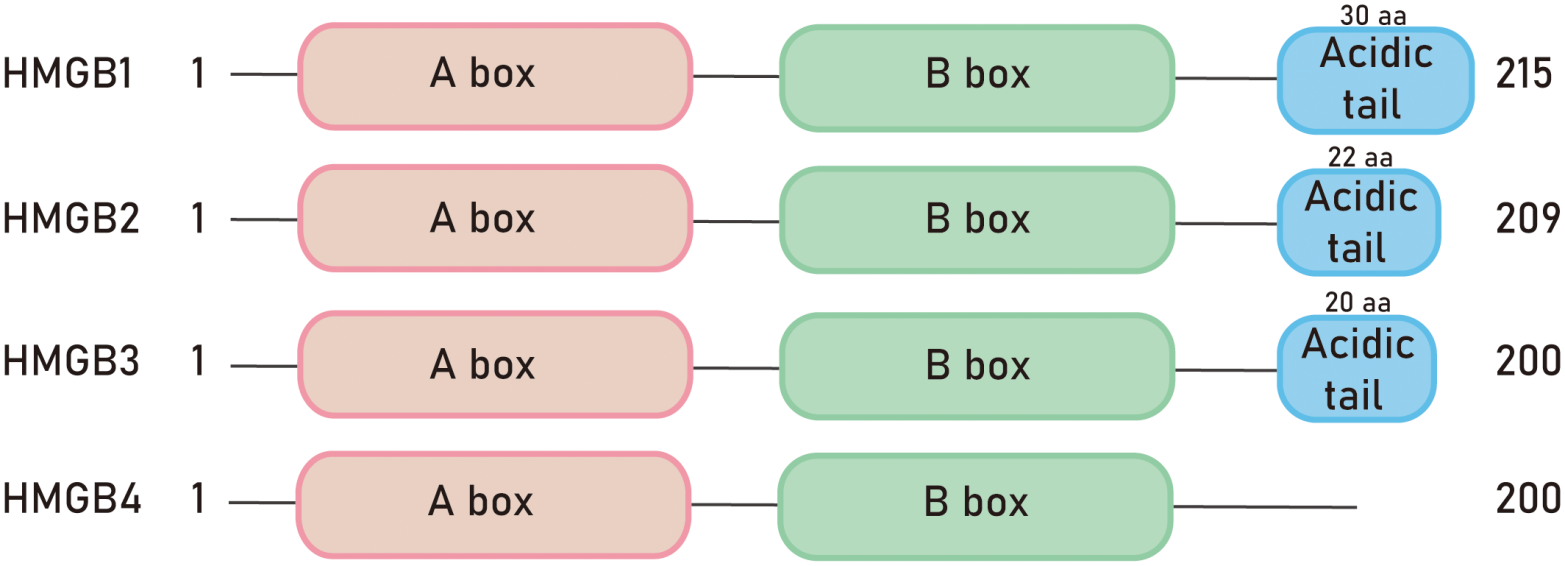
Schematic diagram of HMGB protein family members.

In, 1973, researchers accidentally discovered a group of proteins unrelated to histones during the process of histone separation, which were named non-histone chromosomal protein HMG. These proteins were named after their high electrophoretic mobility in polyacrylamide gels. The HMGB family is the most abundant member of the high mobility group (HMG) superfamily of proteins (1). The HMGB family is the most abundant member of the high mobility group (HMG) superfamily of proteins (2). The proteins in this family are widely expressed and highly conserved in mammals throughout evolution (3). The family includes four members: HMGB1, HMGB2, HMGB3, and HMGB4 (4). All the proteins in the HMGB family have a structural and functional motif called the HMGB domain. Mammalian HMGB proteins consist of two HMG box domains, each composed of about 80 amino acids, and an acidic C-terminal tail (5). However, HMGB4 protein lacks the acidic C-terminal tail (6) (**Figure 1**). The carboxyl-terminal region of HMGB1 contains about 30 consecutive Glu/Asp residues, while HMGB2 and HMGB3 contain about 22 and 20 Glu/Asp residues, respectively (4). The nucleotide sequence conservation of HMGB4 is the lowest among the HMG protein family (7). As nuclear proteins, HMGB proteins are involved in regulating transcription, DNA damage repair, and other nuclear processes (8). Therefore, they play important roles in aging, tumors, autoimmune diseases, inflammation, and other diseases (9). Furthermore, post-translational modifications of HMGB proteins can affect their interactions with DNA and other proteins, as well as their cellular localization (8).

#### 1.1.1 HMGB1

High mobility group box 1 (HMGB1) was first purified from the cell nucleus in the, 1970s and belongs to a typical damage-associated molecular pattern (DAMP) molecule. When cells are stimulated, HMGB1 is released into the extracellular space, acting as an alarm signal (10). The HMGB1 gene encodes a 215-amino acids protein, consisting of a 69-amino acid A box (8-76), an 80-amino acid B box (93-163), and a negatively charged acidic C-terminus. The molecular weight of the protein is 25-30 kDa (11). The A box and B box domains each contain three alpha helices that fold into an L or V shape. The acidic tail at the C-terminus interacts with specific residues in the HMGB1 boxes, regulating the protein’s 3D structure and DNA binding. The acidic tail is an unstructured region of the protein that determines its interaction with histones.

HMGB1 is expressed in almost all eukaryotic cells and is highly conserved in evolution (12). The homology between rat and mouse HMGB1 proteins is 100%, while the amino acid sequence of human and mouse HMGB1 proteins differs by only two amino acids at positions 189 and 202. Specifically, human 189 and 202 are glutamic acid and aspartic acid, respectively, while mouse 189 and 202 are aspartic acid and glutamic acid. Both aspartic acid and glutamic acid side chains contain carboxyl groups, the homology between human and mouse HMGB1 proteins exceeds 99%.

HMGB1 has three subcellular locations: the nucleus, cytoplasm, and cell membrane. Under normal conditions, HMGB1 is mainly found in the cell nucleus, where it binds to chromatin and participates in the formation of nucleoli and protein binding, contributing to the maintenance of nucleolar structure. However, when cells are under stress, HMGB1 translocates from the nucleus to the cytoplasm and is subsequently secreted into the extracellular space (13). It plays a role as a DAMP molecule in guiding inflammation and immune responses (14, 15). Additionally, HMGB1 is involved in DNA transcription, replication, and repair (16). The localization and activity of HMGB1 are influenced by post-translational modifications such as acetylation, methylation, and phosphorylation, which can regulate its translocation and release into the extracellular space under various stress conditions (17, 18).

HMGB1 plays a pivotal role in shaping the immunological landscape of tumorigenesis. In hepatocellular carcinoma (HCC) and gastric cancer (GC), HMGB1 facilitates tumorigenesis by inducing the polarization of macrophages toward an M2 phenotype. In HCC, HMGB1 triggers M2 macrophage polarization through the TLR2/NOX2/autophagy axis, a transformation that promotes tumor growth via upregulation of IL-10 expression (19). In gastric cancer, exosomes originating from gastric cancer cells expressed HMGB1, which prompted M2 macrophage polarization through the inhibition of the NF-κB signaling pathway. This process involves interactions with the transcription factor POU2F1 (20). Overall, current evidence indicates that HMGB1 potentiates the pro-tumorigenic activity of M2 macrophages by interacting with the receptor RAGE (21).

The HMGB1/RAGE axis also profoundly impacts the tumor microenvironment by activating the γδ T cell population. In melanoma, the interaction between HMGB1 and RAGE leads to the accumulation of M2 macrophages, which secrete immunoregulatory factors such as IL-10 (22), thereby suppressing the immune response against tumor. The γδ T cells and cytotoxic lymphocytes have complex roles in tumor immunity. They are also activated by the HMGB1/RAGE pathway, promoting tumor growth and strengthening immunosuppression through the production of cytokines like IL-23 and IL-17. It underscores the central role of the HMGB1/RAGE-IL-23-IL-17-IL-6-Stat3 in the progression of melanoma (23). Furthermore, HMGB1 contributes to anti-tumor immunity through immunogenic cell death (ICD) (24). The release of HMGB1 from dying cells during ICD, a distinct type of cell demise, not only represents the demise of the cell but also elicits an anti-tumor immune response that assists in eradicating tumor cells.

#### 1.1.2 HMGB2

HMGB2 is widely expressed in embryonic stem cells (25), particularly enriched in the thymus, lymphoid organs, and testes of adult mice (4). Knockout mice lacking HMGB2 (*hmgb2-/-*) can survive, but the body weight of male mice decreases (26). HMGB2 is highly homologous to HMGB1 and exhibits high inter-species conservation among mammals. Similar to HMGB1, HMGB2 contains two DNA-binding domains, box A and box B, as well as an acidic C-terminal tail composed of glutamic acid and aspartic acid residues. The main difference between HMGB1 and HMGB2 is that the C-terminal tail of HMGB1 consists of 30 amino acid residues, while HMGB2 has 22 amino acid residues in its C-terminal tail (3). Both HMGB1 and HMGB2 are regulated by post-translational modifications such as acetylation, phosphorylation, glycosylation, and methylation. These modifications can affect the protein’s interactions with DNA/chromatin and regulate its translocation from the nucleus to the cytoplasm and extracellular space (27). HMGB2 plays a similar role to HMGB1 in the development of cardiovascular diseases. In vascular pathologies, HMGB2 has autocrine and paracrine functions and can be released and bind to the membrane molecule RAGE, promoting intracellular signaling (3). Additionally, HMGB2 is involved in cellular aging processes (28) and promotes the proliferation of cervical cancer cells and melanoma cells through various mechanisms (29, 30).

#### 1.1.3 HMGB3

HMGB3 was initially discovered by Marco Bianchi et al. in, 1998 and was found to be highly expressed during embryonic development, while almost undetectable in adult tissues (31). Knockout mice lacking HMGB3 (*hmgb3-/-*) are able to survive, and HMGB3 is crucial for normal development of the eyes and brain (32). HMGB3 contains two HMG boxes and a C-terminal tail composed of 20 amino acids. Early studies have shown that HMGB3 is involved in regulating innate immune activity and the differentiation of normal hematopoietic stem cells (33). In normal adult cells, the expression level of HMGB3 is relatively low, but it is upregulated in tumor tissues such as breast cancer (34), non-small cell lung cancer (35), and glioma (4, 36). HMGB3 is closely associated with tumor occurrence, development, and chemotherapy resistance. In esophageal squamous cell carcinoma, HMGB3 was predominantly localized in the nucleus of tumor cells as determined by immunohistochemical staining, and partially expressed in the cytoplasm. In ovarian cancer, HMGB3 promotes tumor resistance to chemotherapy drugs by regulating DNA damage response pathways (37), and the MAPK/ERK signaling pathway also contributes to the HMGB3-mediated progression of ovarian cancer (38).

#### 1.1.4 HMGB4

In, 2009, a new member of the HMGB (high mobility group box) family called HMGB4 was first discovered by Irwin Davidson et al. HMGB4 is primarily expressed in testicular germ cells and has lower expression in the brain, with no detectable expression in other tissues (31). The molecular weight of HMGB4 protein is 21 kDa, and its main difference from other HMGB members is that it only contains two HMG box domains and lacks the acidic C-terminal tail (6). HMGB4 typically acts as a transcriptional repressor and is encoded by a gene without introns (5). It can tightly bind to DNA, regulate chromatin structure, and participate in the differentiation of neuronal cells. Studies have found that HMGB4 can specifically block the formation of complexes between cisplatin (a commonly used anticancer drug) and DNA, thereby enhancing the sensitivity of testicular germ cell tumor cells to cisplatin (39). In addition, HMGB4 also participates in retinoblastoma (RB)-associated pathways by inhibiting cell cycle and proliferation and enhancing the cytotoxic effects of radiotherapy and cisplatin on cancer cells (40). Currently, the phenotype of HMGB4-deficient mice is not clear, and the biological functions of HMGB4 are largely unknown, requiring further research to reveal its mechanisms and functions in the organism.

HMGB1, a member of the HMGB family, exhibits the highest expression and has been extensively studied. This article focuses on the role and mechanism of HMGB1 and its receptors in tumor development.

### 1.2 The Receptor of HMGB1

#### 1.2.1 TLR2

In the innate immune system, Toll-like receptors (TLRs) are among the key molecules and belong to the pattern recognition receptor (PRR) family. All TLRs share a common synthesis pathway, where they are synthesized in the endoplasmic reticulum (ER), transported to the Golgi apparatus, and eventually localized to the cell surface or different subcellular compartments (41). The expression level of TLR2, its interaction with different ligands, and the types of co-receptors it carries determine its role in mediating pro-inflammatory or anti-inflammatory responses (42). On various cell types including monocytes, tissue-resident macrophages, T cells, and B cells, TLR2 can form homodimers or heterodimers with TLR1 or TLR6 (43). TLR2 is able to recognize endogenous damage-associated molecular patterns (DAMPs) released during infection, tissue necrosis, and injury, such as heat shock proteins and HMGB1. The protein-protein interaction between HMGB1 and TLRs, including TLR2, was first demonstrated by Park et al (44), the binding of TLR2 to HMGB1 is negatively regulated by the C-terminal tail of HMGB1. The HMGB1/TLR2 axis plays a critical role in the pathogenesis of various diseases, including myocardial ischemia/reperfusion injury, peripheral arterial disease, deep vein thrombosis, and lupus nephritis (45, 46). Upon binding, the HMGB1/TLR2 axis plays important roles in cells through signaling pathways such as MyD88-SP1 (47), YAP/HIF-1α (48), and NLRP3-NF-kB (49).

#### 1.2.2 TLR4

TLR4 is a pattern recognition receptor belonging to the TLR family, and it consists of extracellular and intracellular domains (50). TLR4 is widely expressed on the surface of various cells in the human body, including immune cells, intestinal cells, microglia, astrocytes, and neurons. As a multi-ligand receptor, TLR4 can recognize both exogenous pathogen-associated molecular patterns (PAMPs) and endogenous damage-associated molecular patterns (DAMPs), initiating innate immune responses (50). In, 2006, it was demonstrated through fluorescence resonance energy transfer (FRET) experiments and immunoprecipitation that TLR4 can interact with HMGB1 (44). As a receptor, TLR4 binds to the ligand HMGB1 and exerts its biological effects through the 89-108 residues of the HMGB1 B box (51). Once the ligand binds to TLR4, it activates the NF-κB signaling pathway, leading to the production of downstream pro-inflammatory cytokines. This process plays important roles in innate immune defense, protein clearance mechanisms such as autophagy and glial cell phagocytosis, microglial activation, neuronal excitotoxicity, and neurodegeneration (52). The binding of HMGB1 to TLR4 and subsequent NF-κB-mediated cytokine production are strictly regulated by cysteine oxidation-reduction modifications (43). In addition to NF-κB, the HMGB1/TLR4 axis can also impact signaling pathways such as NLRP3-GSDMD (53), MyD88 (54), PI3K/Akt/mTOR (55), and MAPK-p38 (56).

#### 1.2.3 RAGE

The receptor for advanced glycation end products (RAGE) is also an important receptor for HMGB1 protein (57). RAGE was named for its initial discovery as a receptor that can bind to advanced glycation end products (AGEs) (58, 59). In addition to AGEs, RAGE can interact with many endogenous ligands, including S100/calgranulins, HMGB1, amyloid β-protein (Aβ), and exogenous ligands such as lipopolysaccharide (LPS) (60). RAGE and Toll-like receptors may share some ligands (such as HMGB1, S100A8/A9, and LPS) and signaling pathways, and may play similar roles in innate immune responses (61, 62). Before binding to ligands, RAGE assembles into multimers on the cell surface (63, 64); Biochemical analysis has shown that these multimers consist of at least four RAGE molecules, and high cell surface concentrations of RAGE facilitate the formation of multimers (65). Studies have shown that monomeric RAGE has weak affinity for monomeric ligands, hence the formation of multimeric RAGE is necessary for binding to its ligands in a multimeric form (63, 64).

The receptor for advanced glycation end products (RAGE) is composed of 11 exons and is highly conserved at the DNA and protein levels across different mammalian species (66). RAGE is a late-evolving member of the cell surface molecule immunoglobulin superfamily (59, 67) and is located in the major histocompatibility complex (MHC) class III region on chromosome 6 (68), which contains many genes essential for both the innate and adaptive immune systems. RAGE is involved in various diseases, including cardiovascular and neurodegenerative diseases, cancer, and diabetes (69). RAGE is a protein that contains multiple domains, including an extracellular domain (V domain, C1 domain, and C2 domain), a transmembrane domain (a single transmembrane helix), and an intracellular domain (a short cytoplasmic tail) (70). The three main domains are as follows: the first is the hydrophobic extracellular domain present in amino acid residues 23-342. The hydrophobic extracellular domain further refines into three immunoglobulin-like domains, namely the variable domain V (amino acids 23-116), and two constant domains C1 and C2; C1 and C2 are located at amino acids 124-221 and 227-317, respectively. The second domain is the short transmembrane domain (TM) present in amino acid residues 343-363, and the third domain is the cytoplasmic domain present in amino acid residues 364-404. The VC1 domain, which is formed by the integration of the V domain and C1 domain, contains positively charged chemical groups. Some studies have shown that the VC1 domain on RAGE can interact with negatively charged molecules on certain proteins, such as S100/calgranulins, AGEs, HMGB1, and Aβ, to exert specific biological effects (71–73). The binding of HMGB1 to RAGE can activate multiple signaling pathways, including NF-κB, MAPK, MEK, ERK1/2, PI3K, Akt, and TGF-β, which are associated with tumor progression (61, 74–76). Currently, the only known direct binding partner of the cytoplasmic domain of RAGE is mDia1 (17), as identified by yeast two-hybrid screening (77). The crystal structure of RAGE/mDia1 has shown that the arginine at position 366 and the glutamine at position 367 of RAGE interact with the FH1 domain of mDia1 (78).

## 2 HMGB1/RAGE axis and tumor development

### 2.1 HMGB1/RAGE axis and tumor proliferation

Uncontrolled proliferation and unlimited replication are major characteristics of malignant tumors, and inhibiting tumor cell proliferation is necessary to control tumor development. In 13 types of malignant tumors, the HMGB1/RAGE axis has been reported to promote tumor cell proliferation, and several studies have elucidated the signaling pathways involved. Inhibition of cell proliferation can be achieved by blocking cell cycle progression. It has been shown that RAGE is significantly upregulated in hepatocellular carcinoma tissue, and treatment of hepatocellular carcinoma cells (Huh7) with HMGB1 promotes cancer cell progression from the G1 phase to the S phase, accelerating cell division and proliferation (79). Cheng et al. discovered that ethyl pyruvate (EP) (80), a potent inhibitor of HMGB1, inhibits cell cycle progression, and suppresses the growth and proliferation of liver cancer cells by reducing the expression of HMGB1, RAGE, and serine/threonine kinase (AKT) (81). In the study of non-small cell lung cancer, ethyl pyruvate was also found to inhibit the HMGB1/RAGE axis and suppress cell growth through the NF-κB/STAT3 pathway (82).

The HMGB1/RAGE axis has been found to affect proliferation in cervical cancer, glioma, and clear cell carcinoma through the Mitogen-Activated Protein Kinase (MAPK) signaling pathway. He et al. demonstrated through experiments using human cervical cancer HeLa cells and human colon cancer HT29 cells that radiation induces tumor cell necrosis and apoptosis, leading to the passive release of HMGB1. HMGB1 then binds to RAGE receptors through paracrine signaling, activating downstream ERK and p38 signaling pathways and promoting cell proliferation (83). In glioma, it has been reported that HMGB1 can be passively released from dead cells into the extracellular space (84–86). Extracellular HMGB1 interacts with RAGE to stimulate tumor growth (86). Efthalia et al. found that the RAGE/MEK/ERK1/2 signaling pathway mediates HMGB1-induced glioma cell proliferation (87). Grade IV glioblastoma, the most aggressive form of glioma, exhibits unique widespread tissue hypoxia characteristics (88), which can increase HMGB1 expression and extracellular release. Hypoxia-induced HMGB1 activates the RAGE-dependent ERK1/2 signaling pathway, maintaining glioblastoma proliferation, regulating glioma stem cells’ self-renewal, and further promoting tumor progression (89). In clear cell carcinoma, HMGB1 binds to RAGE to initiate intracellular signal transduction and activate ERK, leading to increased cell growth (90). In pancreatic cancer and human breast cancer, the HMGB1/RAGE axis controls cancer cell proliferation through the NF-κB signaling pathway. Priyanka Swami et al. observed that RAGE in pancreatic cancer mice can affect the classical NF-κB signaling pathway by reducing the phosphorylation levels of p65. In human pancreatic cancer cells and tumors, it has been demonstrated that both RAGE ligands, HMGB1 and S100P, stimulate RAGE. However, the animal model used by the authors does not express functional S100P, suggesting that HMGB1 activation of RAGE can stimulate pancreatic cancer cells and promote their proliferation (91). Lan et al. showed that in human breast cancer cells, quercetin inhibits cell survival and proliferation by protecting against cell death, reducing the expression of HMGB1 and RAGE, inhibiting p65 nuclear translocation, and suppressing NF-κB activation (92).

Two articles have demonstrated that the HMGB1/RAGE axis can promote proliferation in nasopharyngeal carcinoma (NPC). In colorectal cancer and gastric cancer, the HMGB1/RAGE axis also plays a role in tumor promotion. In, 2015, a study showed for the first time that knockdown of HMGB1 inhibited the activation of the HMGB1/RAGE axis, downregulated the expression of p-ERK1/2, and suppressed the proliferation of NPC cells (93). In a study on NPC in, 2016, it was reported that after stimulation with Epstein-Barr virus (EBV), HMGB1 was significantly upregulated in the cytoplasm in a dose-dependent manner, while it was significantly downregulated in the nucleus. The levels of HMGB1 in the supernatant increased significantly, and RAGE was also significantly upregulated, leading to accelerated proliferation of NPC cells. Zhu et al. demonstrated that the pro-proliferative effect of HMGB1 in NPC cells is RAGE-dependent (94). In colorectal cancer, the overexpression of HMGB1 and RAGE signaling may activate the transcription factor Yes-associated protein 1 (Yap1) through direct interaction with K-Ras, thereby promoting the proliferation and stemness of colorectal cancer cells (95). The HMGB1/RAGE axis regulates gastric cancer cell proliferation through the Akt/mTOR and ERK signaling pathways (96). Consistent with previous reports (97, 98), the expression levels of HMGB1 in gastric cancer tissues and cells were higher than those in adjacent lung tumor tissues and normal gastric epithelial cells (96). Tang et al. found that overexpression of HMGB1 in gastric cancer cells increased RAGE expression, but not TLR2 and TLR4. The expression levels of cyclin D1, cyclin E1, and proliferating cell nuclear antigen (PCNA) were elevated, and the cell proliferation ability was enhanced (96).

**Figure 2:**
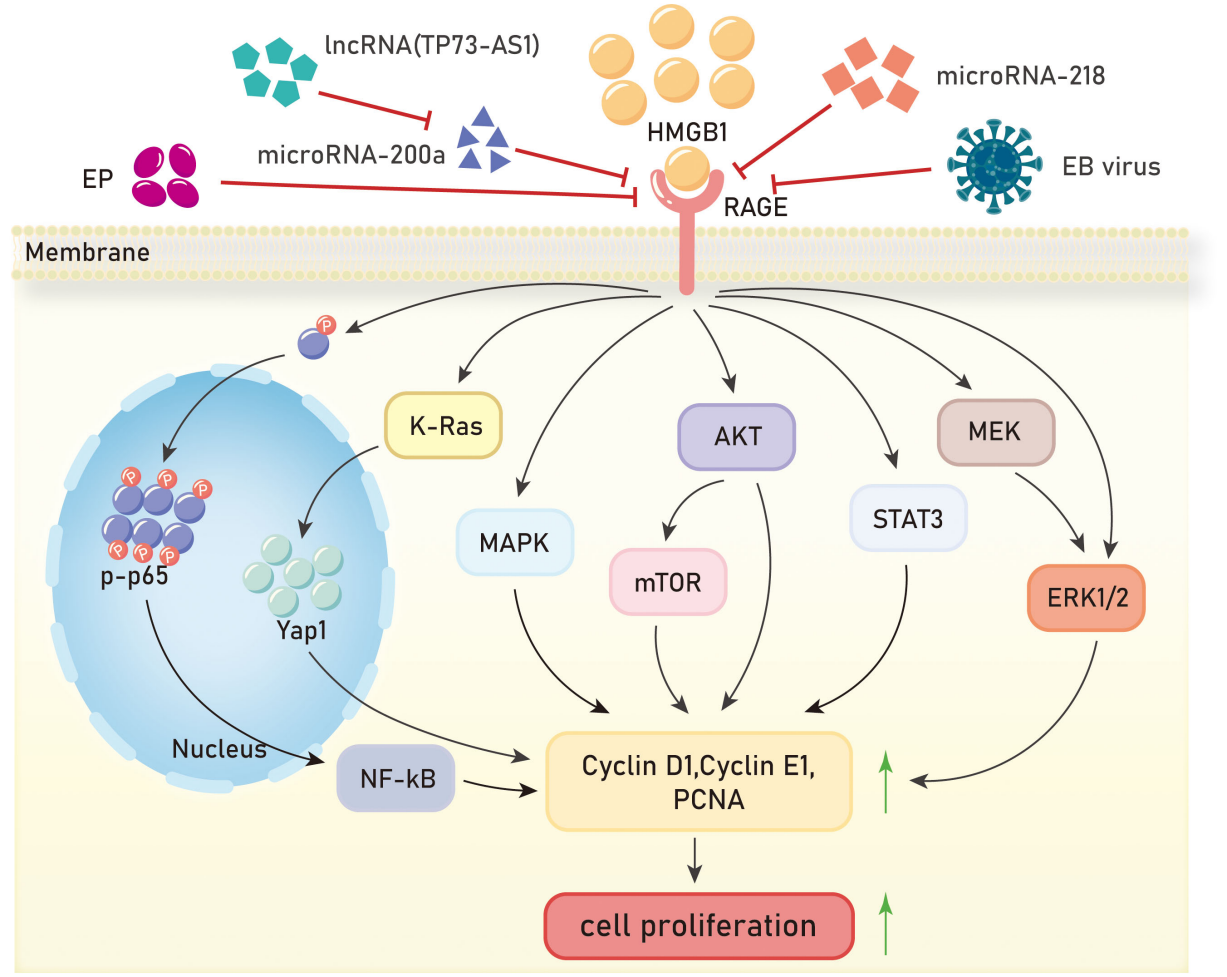
Mechanisms of HMGB1/RAGE axis in tumor proliferation. EP, ethyl pyruvate;EB virus,Epstein-Barr virus;NF-kB, Nuclear factor kappa-B;K-Ras, Kirsten-rat sarcoma viral oncogene homolog;Yap1,Yes-associated protein 1;MAPK, Mitogen-activated protein kinase;AKT, Protein kinase B;mTOR, mammalian target of rapamycin;STAT3, Signal Transducer And Activator Of Transcription 3;MEK, Mitogen-activated protein kinase kinase;ERK1/2, extracellular regulated protein kinases.

Functional RNAs, including long non-coding RNAs (lncRNAs) and endogenous non-coding RNAs (microRNAs or miRNAs), have been found to regulate the HMGB1/RAGE axis and affect proliferation in liver cancer and glioblastoma. Many lncRNAs have complementary sequences with protein-coding genes, suggesting that they may play a role in mRNA splicing, editing, transport, translation, and degradation. One such lncRNA, TP73-AS1, targets miR-200a to inhibit its expression, thereby upregulating HMGB1/RAGE expression and promoting proliferation in liver cancer cells (99). Gu et al. discovered that microRNA-218 inhibits glioblastoma cell proliferation by negatively regulating the HMGB1-RAGE axis (3) (**Figure 2**).

The HMGB1/RAGE axis promotes tumor cell proliferation in various malignant tumors, including hepatocellular carcinoma, non-small cell lung cancer, cervical cancer, glioma, clear cell renal cell carcinoma, pancreatic cancer, breast cancer, nasopharyngeal carcinoma, colorectal cancer, and gastric cancer. Additionally, the role and mechanism of the HMGB1/RAGE axis in promoting tumor proliferation in other tumor types such as osteosarcoma, lung cancer, and thyroid cancer remain to be elucidated. The interaction between HMGB1 and its receptor RAGE promotes cell proliferation through cell cycle progression, MAPK, NF-κB, Akt/mTOR, ERK, and other signaling pathways. Certain functional RNAs, including long non-coding RNAs and microRNAs, regulate the expressions of HMGB1/RAGE and impact tumor cell proliferation. However, there are also some functional RNAs have the potential to inhibit HMGB1/RAGE axis, representing a promising avenue for research. Furthermore, the development of molecular drugs that inhibit the HMGB1/RAGE axis is crucial for cancer therapy.

### 2.2 HMGB1/RAGE axis and Tumor

#### 2.2.1 Apoptosis

Cell apoptosis is a fundamental biological phenomenon that plays a crucial role in the evolution of organisms, maintenance of internal environment stability, and development of multiple systems. It also plays an important role in inhibiting tumor development. Numerous studies have revealed a significant association between the HMGB1/RAGE axis and cell apoptosis in tumors. Cell apoptosis, known as programmed cell death, is typically triggered by excessive DNA damage that cannot be adequately repaired. Currently, gemcitabine (GEM) (100) is the most widely used cytotoxic drug for treating pancreatic cancer in clinical settings. DNA damage induced by gemcitabine can lead to cell apoptosis (101). In a mouse model of pancreatic cancer treated with gemcitabine, inhibition of RAGE enhances gemcitabine-induced cell apoptosis. Hence, RAGE is believed to play a role in inhibiting cell apoptosis in pancreatic cancer (91). Silencing RAGE in prostate tumor cells results in decreased HMGB1 expression and increased expression of death receptors DR4 and DR5, demonstrating that disruption of the HMGB1-RAGE axis induces apoptosis in prostate tumors (102). Inhibition of the HMGB1-RAGE axis in pancreatic cancer cells also induces cell apoptosis (9). In another study on pancreatic tumors, downregulation of RAGE expression increases caspase-3 activity and enhances tumor cell apoptosis. Conversely, when RAGE is present, it restricts the translocation of p53 to mitochondria, thereby inhibiting cell apoptosis. Experiments have also demonstrated that silencing HMGB1 increases the expression of cleaved PARP, an apoptosis marker, in pancreatic cancer cells with downregulated RAGE expression, further promoting apoptosis (72).

Multiple small chemical molecules have been reported to have anti-tumor effects and promote tumor cell apoptosis. 4-acetylquinone B (4-AAQB) is an ubiquitin-ketone derivative from the Formosan camphor tree, known for its anti-inflammatory and antioxidant abilities (103). In pancreatic cancer cells treated with 4-AAQB, there is a significant increase in Bax, a significant decrease in Bcl-xL, and a significant increase in the Bax/Bcl-xL ratio, promoting pancreatic cancer cell apoptosis (104). Aloin (ALO), extracted from aloe vera, is a bioactive compound with anti-tumor effects that can induce apoptosis in lung cancer, colorectal cancer, and breast cancer cells (105, 106). Tao et al. demonstrated that ALO can reduce the expression levels of HMGB1 and its receptor RAGE, inhibiting the release of HMGB1. In breast cancer cells with silenced HMGB1, ALO can suppress the activation of the Akt-mTOR-P70S6K and ERK-P90RSK-CREB signaling pathways induced by HMGB1 (107).

**Figure 3:**
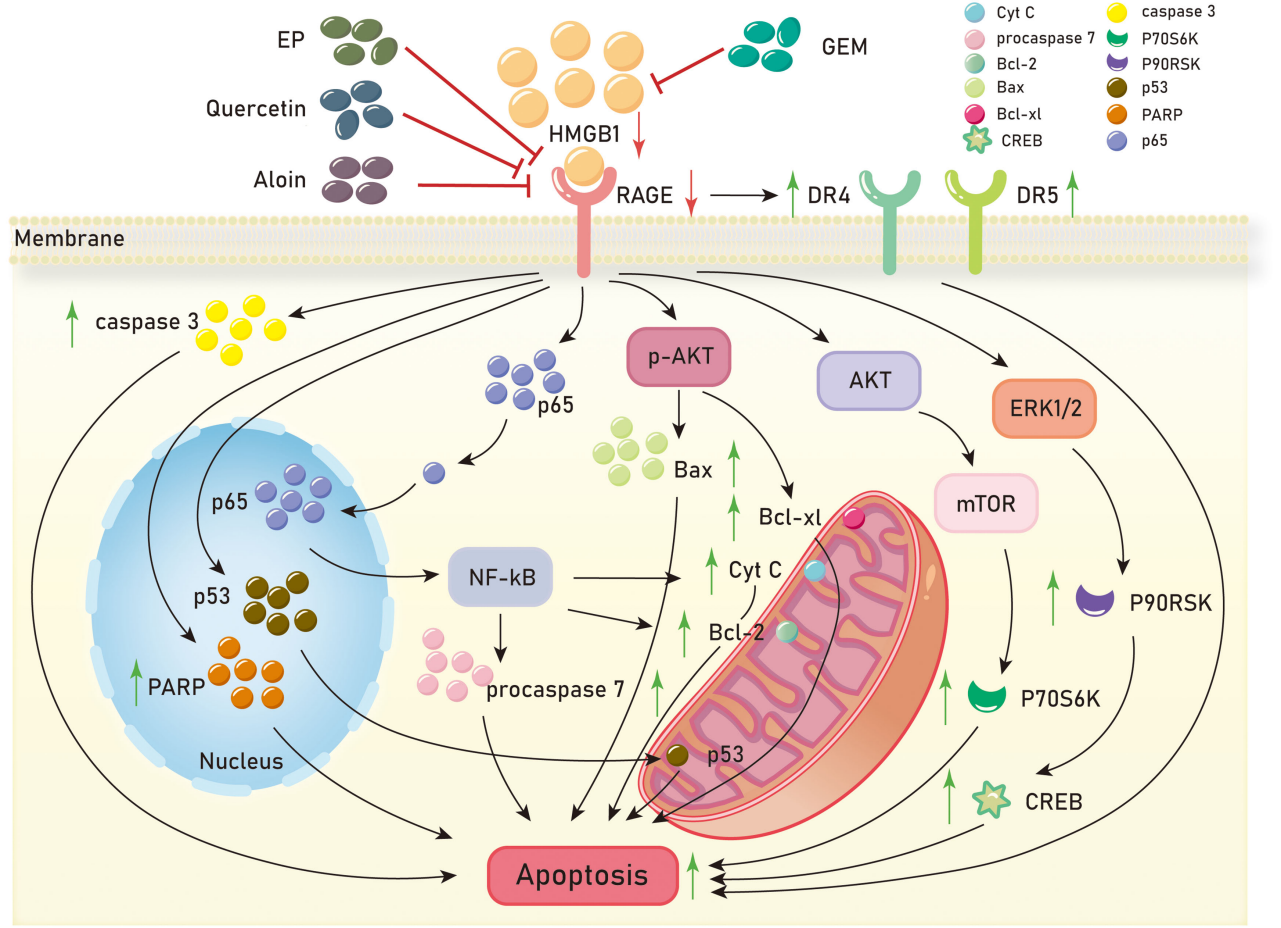
Mechanisms of HMGB1/RAGE axis in tumor apoptosis. EP, ethyl pyruvate;GEM, gemcitabine;DR4, death receptors 4;DR5, death receptors 5;NF-kB, Nuclear factor kappa-B;PARP, Poly ADP-ribose polymerase;AKT, Protein kinase B;mTOR, mammalian target of rapamycin;ERK1/2, extracellular regulated protein kinases 1/2;P70S6K, p70 ribosomal protein S6 kinase;P90RSK, p90 ribosomal protein S6 kinase;CREB, cAMP -response element binding protein.

Two anti-cancer drugs, quercetin and lucidone A, inhibit the HMGB1/RAGE axis to promote apoptosis in breast cancer and pancreatic cancer, respectively. Ethyl pyruvate (EP), an inhibitor of HMGB1, enhances apoptosis in liver cancer cells. Quercetin, a well-known anti-cancer agent, is a natural flavonoid that inhibits the expression of HMGB1 and RAGE in human breast cancer at the transcription and translation levels. Treatment with quercetin in breast cancer MCF7 cells increases cytoplasmic p65 expression, inhibiting its nuclear translocation and thus suppressing NF-kB activation. Additionally, quercetin treatment downregulates the levels of mitochondrial cytochrome C, procaspase-7, and the anti-apoptotic protein Bcl-2. These findings suggest that quercetin induces apoptosis through the inhibition of the HMGB1-RAGE axis (92). Ethyl pyruvate (EP), a simple ester derived from pyruvic acid, is an effective inhibitor of HMGB1 and can disrupt the AKT pathway (108). In liver cancer, EP inhibits the expression of HMGB1 and RAGE. EP reduces p-AKT expression through the HMGB1-RAGE axis and increases the Bax/Bcl-2 ratio, promoting apoptosis in liver cancer (81). Lucidone, a naturally occurring cyclopentenedione analogue, extracted from the fruit of Lindera erythrocarpa Makino, a plant widely distributed in Asia, has been shown to exhibit anti-inflammatory effects through the mediation of NF-κB and MAPK signaling pathways (109). Experimental results indicate that lucidone reduces the levels of the anti-apoptotic protein Bcl-xL and increases the ratio of the pro-apoptotic protein Bax/Bcl-xL in human pancreatic cancer MIA Paca-2 cells. Many patients with pancreatic ductal adenocarcinoma (PDAC) develop resistance to gemcitabine (GEM). Further experiments reveal that lucidone significantly inhibits the protein levels of HMGB1 and RAGE in GEM-resistant pancreatic cancer cells. In conclusion, lucidone promotes apoptosis in human pancreatic cancer cells through the HMGB1-RAGE axis (110) (**Figure 3**).

Collectively, the HMGB1/RAGE axis has been found to be closely related to tumor cell apoptosis, and several small-molecule chemical drugs have been reported to have anti-tumor effects by promoting tumor cell apoptosis via HMGB1/RAGE axis. These drugs include 4-acetylquinoline B, aloin, quercetin, ethyl pyruvate, and ganoderone. These studies suggest that the HMGB1/RAGE axis plays an important role in tumor cell apoptosis, and these related drugs have therapeutic potential in tumor treatments. Future research can further explore the molecular mechanisms of the HMGB1/RAGE axis in tumor cell apoptosis, search for more anti-tumor drugs, and investigate their relationship with the HMGB1/RAGE axis. In addition, personalized therapy for tumors is also a research hotspot, and further research can explore the different responses of patients to anti-tumor drugs, as well as the role of the HMGB1/RAGE axis in this process. Overall, the relationship between the HMGB1/RAGE axis and tumor cell apoptosis provides new ideas and potential targets for tumor treatment, but further research is needed to deepen our understanding of its mechanisms and develop more effective treatment strategies.

### 2.3 HMGB1/RAGE axis and tumor migration, invasion, EMT

Metastasis is the main cause of death in most cancer patients, posing a significant challenge in the fight against cancer. Metastasis in tumor cells is characterized by migration, invasion, and epithelial-mesenchymal transition (EMT). In the growth processes of liver cancer, colon cancer, colorectal cancer, cervical cancer, prostate cancer, and pancreatic cancer, the HMGB1/RAGE axis can promote tumor migration and invasion through the NF-kB signaling pathway. In the liver cancer cell line HCCLM3, overexpression of HMGB1 and RAGE is observed, and inhibition of the HMGB1/RAGE axis reduces the expression of p50 and p65 in the NF-kB signaling pathway, thereby inhibiting the migration and invasion of HCCLM3 (111). In, 2013, it was reported that the addition of exogenous HMGB1 to H22 liver cancer cells, acting on RAGE and through the NF-kB pathway, increases the expression of matrix metalloproteinase 9 (MMP9), promoting the invasion of H22 cells (112). In the colon cancer cell line LoVo, translationally controlled tumor protein (TCTP) upregulates the expression and secretion of HMGB1. Experimental evidence suggests that the HMGB1/RAGE axis mediates the NF-kB signaling pathway, promoting the invasion of LoVo cells *in vitro* and the metastasis of colon cancer *in vivo* (113). Pang et al. found that inhibition of the HMGB1/RAGE/Snail/NF-κB signaling pathway can reverse HMGB1-induced EMT in colorectal cancer cell line CRC, thereby preventing the migration and invasion of CRC cells (114). In, 2016, an article reported that HMGB1 may be involved in the invasion, migration, and EMT of cervical cancer cells by activating the NF-kB signaling pathway through binding to RAGE (115). Zhang et al. suggested that HMGB1 promotes EMT in prostate cancer PC3 cells by activating the RAGE/NF-κB signaling pathway, upregulating the expression of EMT markers MMP-1, MMP-3, and MMP-10, and promoting prostate cancer metastasis (116). Studies have shown that HMGB1 activates RAGE to affect the NF-κB signaling pathway by reducing p65 phosphorylation levels, promoting pancreatic cancer metastasis, and inducing chemoresistance to gemcitabine (91).

In glioblastoma, clear cell renal cell carcinoma, gastric cancer, nasopharyngeal carcinoma, melanoma, and non-small cell lung cancer, the HMGB1/RAGE axis plays a role in tumor metastasis through the MAPK signaling pathway. When human glioblastoma cells undergo necrosis, HMGB1 is released into the extracellular environment, where it can act on adjacent cells to promote tumor progression. HMGB1 regulates the migration of T98G glioblastoma cells through the RAGE/MEK/ERK signaling pathway (86). The binding of HMGB1 to RAGE initiates intracellular signaling and activates ERK, leading to increased migration and invasion of clear cell renal cell carcinoma cells (RCCC) (90). In gastric cancer cells, HMGB1 acts on RAGE to enhance Akt/mTOR and ERK signaling pathway phosphorylation, promoting cell migration (96). Knockdown of HMGB1 in nasopharyngeal carcinoma cells inhibits the activation of the HMGB1/RAGE pathway, downregulating the expression of p-ERK1/2 and reducing the migration and invasion capabilities of cancer cells (93). Glycyrrhizin improves lung metastasis in melanoma, and further experiments show that it weakens NF-KB and ERK1/2 expression by acting on the HMGB1/RAGE axis, inhibiting melanoma lung metastasis (117). In, 2019, it was reported that ethyl pyruvate (EP) reduces MMP9 levels and attenuates the migration and invasion of non-small cell lung cancer (NSCLC) cells by inhibiting the HMGB1/RAGE axis (82).

The HMGB1/RAGE axis is involved in the metastasis of breast cancer, chondrosarcoma, and prostate cancer through the PI3K-AKT-mTOR signaling pathway, respectively. Research has shown that fibroblasts in breast cancer activate HMGB1 release into the tumor microenvironment, where it interacts with RAGE and promotes invasion of breast cancer cells and the expression of programmed death ligand 1 (PD-L1) through the PI3K/AKT signaling pathway (118). The HMGB1/RAGE axis enhances the expression of integrin α5β1 in chondrosarcoma cells and promotes their migration through the PI3K/Akt/c-Jun/AP-1 signaling pathway (119). Verbascoside inhibits TGF-β and the EMT process through the HMGB1/RAGE axis, thereby reducing cell proliferation and invasiveness in prostate cancer via the PI3K/AKT/mTOR pathway (120). In gastric cancer, prostate cancer, rhabdomyosarcoma, breast cancer, and hypopharyngeal cancer, the HMGB1/RAGE axis is involved in the migration, invasion, and EMT of these cancers. Clinical evidence suggests that high expression of HMGB1 and RAGE is associated with a significant decrease in the five-year survival rate in gastric cancer patients with diabetes compared to those with low expression (96). mRNA expression of HMGB1 and RAGE is upregulated in prostate cancer tissues, and increased RAGE expression induces the invasive ability of prostate cancer (121). Francesca et al. concluded that HMGB1 activates RAGE in rhabdomyosarcoma cells via autocrine signaling. Forced expression of RAGE in RAGE-negative rhabdomyosarcoma cells TE671 resulted in decreased invasive ability (122). The secretion of HMGB1 in breast cancer cells is positively correlated with their metastatic potential. Further experiments have shown that HMGB1 secretion in breast cancer cells promotes fibroblast activation, which, through RAGE, upregulates aerobic glycolysis and promotes the metastasis of breast cancer cells (123). Li et al. demonstrated that HMGB1 can attenuate TGF-β-induced invasion, migration, and epithelial-mesenchymal transition in hypopharyngeal cancer cells FaDu by regulating RAGE expression (124).

**Figure 4:**
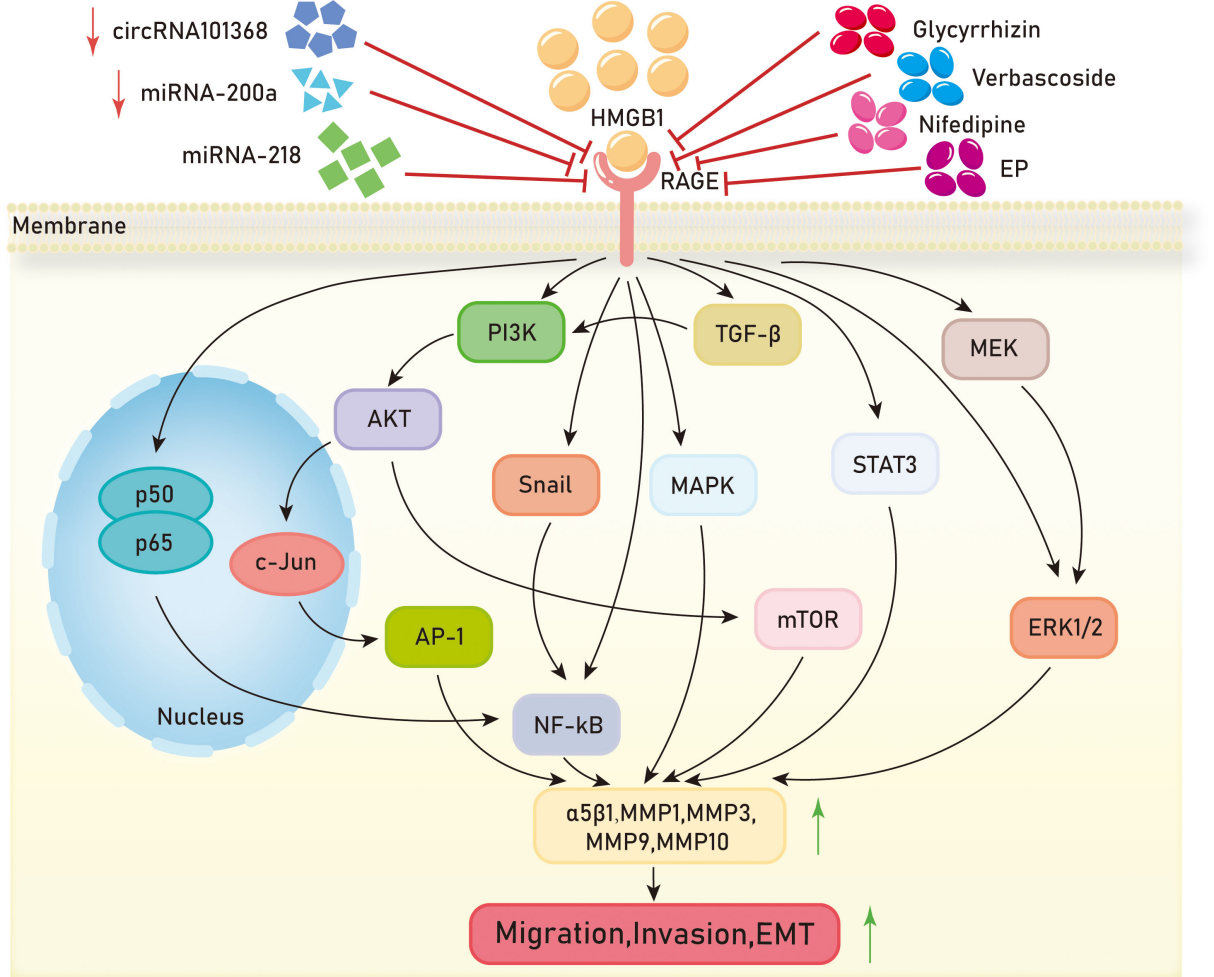
Mechanisms of HMGB1/RAGE axis in tumor migration,invasion and EMT. EP,ethyl pyruvate;PI3K,Phosphatidylinositol-3-kinase;AKT,Protein kinase B;AP-1,activator protein-1;NF-kB, Nuclear factor kappa-B;MAPK, Mitogen-activated protein kinase;mTOR, mammalian target of rapamycin;STAT3, Signal Transducer And Activator Of Transcription 3;MEK, Mitogen-activated protein kinase;ERK1/2, extracellular regulated protein kinases 1/2;MMP,Matrix metalloproteinases.

Recent studies have revealed the involvement of small RNAs and chemical substances in the HMGB1/RAGE axis. In hepatocellular carcinoma (HCC) cell line HCC, the decreased expression levels of circRNA, 101368 and miR-200a inhibit HCC migration. The circRNA, 101368/miR-200a axis regulates HCC migration through the HMGB1/RAGE signaling pathway (115). The interaction between HMGB1 and RAGE enhances the migration activity of human squamous cell carcinoma SCC7 cells, while nifedipine dose-dependently inhibits the HMGB1-RAGE interaction in SCC cells (125). MIR-218 may negatively regulate the HMGB1/RAGE axis by targeting HMGB1, thereby inhibiting the invasion of glioblastoma cells (**Figure 4**).

Overall, the HMGB1/RAGE axis is involved in the migration, invasion, and metastasis of various cancers. It regulates the epithelial-mesenchymal transition (EMT) process of cells through different signaling pathways, including NF-kB, MAPK, PI3K/AKT/mTOR, etc., promoting cancer cell metastasis. High levels of HMGB1 and RAGE expression in tumors are associated with poor prognosis, suggesting a role of HMGB1/RAGE axis in tumor prognosis. Although many chemical drugs and small RNAs have been found to regulate the HMGB1/RAGE axis and inhibit tumor cell metastasis and invasion, their research is still in the early stage and further studies are needed to validate their efficacy and safety. Additionally, future research is required to explore the exact molecular mechanisms of the HMGB1/RAGE axis in tumor cell apoptosis and search for more drugs having the potential to regulate HMGB1/RAGE axis. Furthermore, an essential avenue is personalized therapy, developing treatment strategies tailored to individual variances, aiming to enhance treatment effectiveness while minimizing side effects. In summary, additional investigation is warranted to comprehend the involvement and regulatory mechanisms of the HMGB1/RAGE axis in tumor metastasis, which will offer novel insights and approaches for advancing tumor treatment.

### 2.4 HMGB1/RAGE axis and tumor autophagy

Autophagy, also known as self-eating, is a process in which eukaryotic cells utilize lysosomes to degrade their own cytoplasmic proteins and damaged organelles under the regulation of autophagy-related genes (ATGs). Autophagy serves as a cellular self-protective mechanism, preventing cell damage and promoting cell survival under conditions of nutrient deprivation. It plays a beneficial role in cell growth and development, protecting cells from metabolic stress and oxidative damage, and maintaining cellular homeostasis, as well as the synthesis, degradation, and recycling of cellular products (126). During the early stages of tumorigenesis, autophagy can prevent tumor formation and inhibit cancer progression. However, as tumors advance and face environmental pressures, autophagy, as a dynamic degradation and recycling system, contributes to the survival and growth of established tumors, promoting cancer invasiveness by facilitating metastasis (127).

Currently, research on autophagy in pancreatic cancer is relatively comprehensive. The HMGB1/RAGE axis has been shown to promote autophagy in pancreatic cancer and contribute to its progression in multiple studies. In autophagy regulation, HMGB1 is a multifunctional protein with location-dependent functions. Under normal conditions, HMGB1 is located in the cell nucleus, but under stress conditions, it can translocate to the cytoplasm or be released into the extracellular space (128). Reduced HMGB1 promotes cancer cell autophagy, while oxidized HMGB1 promotes cancer cell apoptosis. After reduction, HMGB1 is released by cancer cells into the extracellular space, where it interacts with RAGE, inducing Beclin-dependent autophagy (129). As a cellular defense mechanism, cytoplasmic HMGB1 directly interacts with Beclin 1 (130). Antioxidant enzymes, such as superoxide dismutase, and small molecule antioxidants, such as N-acetyl-l-cysteine, inhibit HMGB1 activation and pancreatic autophagy induction (131). Inhibition of the HMGB1-RAGE axis leads to reduced autophagy in pancreatic cancer and inhibits its progression. In pancreatic cancer, RAGE-mediated autophagy acts by reducing the phosphorylation level of mammalian target of rapamycin (mTOR) and limiting the formation of the Beclin-1/VPS34 autophagy complex. Exogenous rhHMGB1 can promote autophagy in pancreatic cancer cell lines through RAGE-dependent signaling pathways (132). Clinical evidence shows that pancreatic cancer patients develop chemoresistance and cytotoxic effects to gemcitabine (GEM) chemotherapy related to autophagy (133). Bax can directly inhibit autophagy (134), and treatment of pancreatic cancer cells with 4-AAQB enhances the expression of pro-apoptotic protein Bax and reduces the expression levels of autophagy-related proteins (Atg5, Beclin-1, and LC3 II). Chen et al. demonstrated that 4-AAQB downregulates autophagy through inhibition of the HMGB1/RAGE-initiated PI3K/Akt/MDR1 signaling pathway (104). They also showed that lucidone combined with gemcitabine reduces autophagy and promotes cell apoptosis through the HMGB1/RAGE/PI3K/Akt signaling pathway, thereby slowing down the development of pancreatic cancer (110). Currently, besides surgery, the main treatment methods for patients with exocrine pancreatic cancer in clinical practice are radiotherapy and chemotherapy, which can significantly activate autophagy in pancreatic cancer cells (135, 136). Compared to other epithelial cancers, pancreatic cancer typically exhibits high basal levels of autophagy, which is associated with poor prognosis in patients (137).

**Figure 5:**
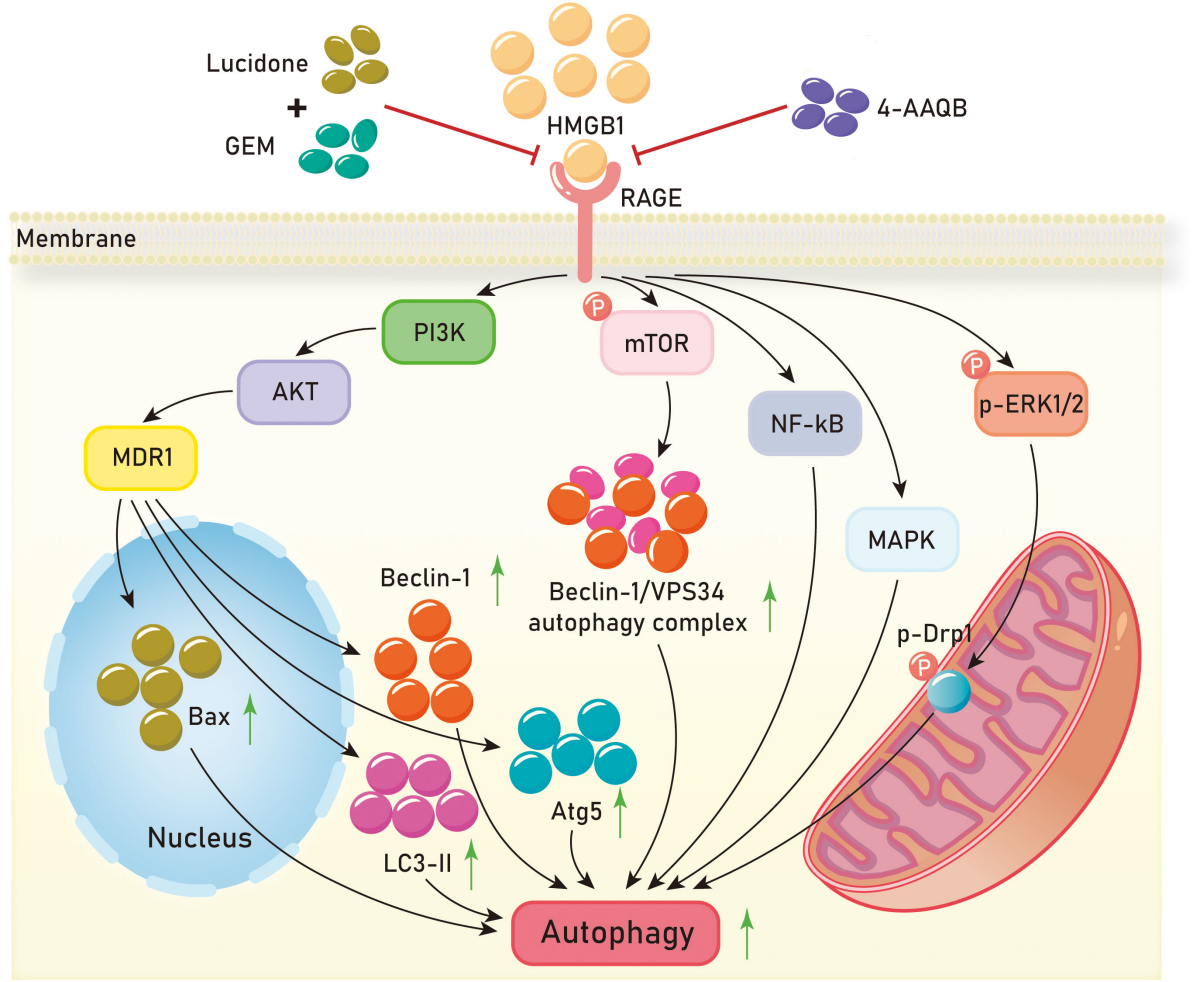
Mechanisms of HMGB1/RAGE axis in tumor autophagy. GEM,gemcitabine;4-AAQB,4-acetylquinone-B;PI3K,Phosphatidylinositol-3-kinase;AKT,Protein kinase B;MDR1, Multi-drug Resistance-1;mTOR, mammalian target of rapamycin;NF-kB, Nuclear factor kappa-B;MAPK, Mitogen-activated protein kinase;ERK1/2, extracellular regulated protein kinases 1/2;Drp1, dynamin-related protein 1.

In addition to pancreatic cancer, increased autophagy mediated by the HMGB1/RAGE axis has been observed in renal cell carcinoma, colorectal cancer, and clear cell renal cell carcinoma. Knocking down HMGB1 in renal cell carcinoma significantly inhibits the expression of RAGE, as well as the expression of autophagy proteins LC3II and Beclin1. The HMGB1-RAGE axis mediates autophagy in renal cancer cells (138). Huang et al. found that both HMGB1 and RAGE are significantly upregulated in colorectal cancer tissues. Experimental evidence has shown that in colorectal cancer cells, extracellular HMGB1 activates ERK1/2 through its interaction with RAGE. The HMGB1-RAGE axis activates the phosphorylation of dynamin-related protein 1 (Drp1) (139, 140) leading to mitochondrial fission and cell autophagy, which promotes chemotherapy resistance and tumor growth in colorectal cancer (141). HMGB1, RAGE, and autophagy proteins LC3, Beclin-1, and PI3K are significantly increased in clear cell renal cell carcinoma. Experimental evidence has demonstrated that the interaction between HMGB1 and RAGE initiates signaling pathways such as ERK1/2 phosphorylation, NF-kB, and MAPK, thereby promoting autophagy in ccRCC (142) (**Figure 5**).

These investigations suggest that the activation of the HMGB1/RAGE axis can stimulate autophagy in tumor cells by elevating the expression of autophagy-related proteins LC3 and Beclin-1, achieved through the activation of signaling pathways like PI3K/Akt, MAPK, and NF-κB. Furthermore, the distinct roles played by the reduced and oxidized states of HMGB1 in tumor cells necessitate further exploration. Subsequent studies should probe into the autophagy regulatory mechanisms governed by the HMGB1/RAGE axis in tumors and explore additional therapeutic approaches targeting this axis. Additionally, research efforts should investigate the interplay between the HMGB1/RAGE axis and other signaling pathways for a more comprehensive understanding of the regulatory network governing autophagy in tumor cells.

### 2.5 HMGB1/RAGE axis and tumor angiogenesis

The process of angiogenesis is primarily initiated by the tumor itself (143). As a malignant tumor grows to a certain size, it leads to cellular hypoxia. Hypoxia is a crucial trigger for tumor angiogenesis (144). Hypoxia causes an increased expression of angiogenic molecules, including growth factors, cytokines, bioactive lipids, and matrix-degrading enzymes, in hypoxic cells. These molecules bind to receptors on adjacent vascular endothelial cells to initiate the formation of new blood vessels (145). Once tumor neovascularization occurs, an adequate vascular system and blood supply continue to provide oxygen and nutrients to cancer cells, thereby promoting tumor growth, progression, and metastasis (145).

In several types of tumors, including renal cell carcinoma, oral squamous cell carcinoma, laryngeal squamous cell carcinoma, and colorectal cancer, angiogenesis is closely associated with malignant tumor progression. Experimental evidence has shown that inhibiting the HMGB1/RAGE axis can suppress tumor angiogenesis. HMGB1 and RAGE are overexpressed in glioblastoma, and the impact of RAGE ablation in the tumor microenvironment (TME) on glioblastoma growth appears to be due to reduced tumor inflammation and impaired angiogenesis (146). Knocking down RAGE in glioblastoma leads to decreased inflammation and tumor angiogenesis, inhibiting tumor development. In renal cell carcinoma cell lines, reducing the expression of HMGB1 and RAGE significantly decreases the expression of vascular endothelial growth factors (VEGF) and its receptor VEGFR2. The HMGB1/RAGE axis regulates angiogenesis in renal cell carcinoma (138). Late-stage oral squamous cell carcinoma (OSCC) is mainly characterized by local invasion and lymph node metastasis (147), and angiogenesis is one of the main factors contributing to OSCC progression (148). The expression of RAGE in OSCC tumor tissues is significantly correlated with VEGF, and experimental evidence has shown that HMGB1 induces VEGF secretion in OSCC cell lines HSC-3 and HSC-4 (149). A study on laryngeal squamous cell carcinoma (LSCC) has shown that HMGB1 increases lymphangiogenesis by activating RAGE on M2 macrophages (150). For many LSCC patients, the main cause of death is tumor dissemination, and lymph node metastasis is early evidence of tumor dissemination (151). All-thiol HMGB1 (at-HMGB1) binds to its receptor RAGE, promoting the secretion of the angiogenic factor VEGF, thereby facilitating angiogenesis in colorectal cancer patients. The HMGB1/RAGE axis is an important target for treating tumor angiogenesis (152).

Tumor endothelial cells (ECs) secrete HMGB1 and increase the expression of RAGE, promoting the angiogenic capacity of tumor ECs. Evodiamine (EVO), a bioactive compound derived from the plant Evodia rutaecarpa, effectively inhibits the HMGB1/RAGE pathway. In a study conducted in, 2013, it was found that activated ECs secrete HMGB1, which stimulates the migration and angiogenic capacity of ECs *in vitro* and *in vivo* through autocrine or paracrine mechanisms. *In vivo* experiments using HMGB1 antibodies successfully targeted and inhibited tumor angiogenesis in the chorioallantoic membrane (CAM) of chick embryos. *In vitro* experiments demonstrated that exogenous HMGB1 increased the expression of RAGE and TLR4 in ECs, further confirming that RAGE is the primary receptor for HMGB1 in tumor ECs (153). In, 2021, Ren et al. predicted through bioinformatics that EVO, a bioactive compound from Evodia rutaecarpa, could interact with RAGE or its major ligands. Previous studies have reported that EVO exhibits inhibitory effects on tumor proliferation and promotes apoptosis in various studies (154, 155). Subsequent *in vitro* and *in vivo* experiments demonstrated that EVO inhibits the growth and angiogenesis of oral squamous cell carcinoma (OSCC) and is associated with the HMGB1/RAGE axis. The authors suggest that EVO directly binds to HMGB1 and may also participate in protein degradation. It can reduce the activity of the RAGE pathway by influencing the binding of HMGB1 to RAGE (156).

Hypoxia prompts the expression of angiogenic molecules within tumor cells, thereby instigating the development of new blood vessels. Angiogenesis is intricately linked to the progression of diverse malignancies, such as renal cell carcinoma, oral squamous cell carcinoma, laryngeal squamous cell carcinoma, and colorectal cancer. The HMGB1/RAGE axis plays a crucial role in regulating angiogenesis, and its inhibition has been demonstrated to curb tumor angiogenesis and progression. Rutaecarpine, the active compound in Evodia rutaecarpa, has been identified as a suppressor of the HMGB1/RAGE pathway, exhibiting the potential to hinder tumor growth and angiogenesis. Future investigations should delve deeper into the regulatory mechanisms of this axis and strive to develop more effective strategies for addressing tumor angiogenesis, offering valuable contributions to scientific publications.

## 3 Conclusion

Due to the various subcellular localization of HMGB1 and its interaction with diverse receptors, its role in tumors is complex. HMGB1 can be actively released or passively secreted into the extracellular space in tumor cells, where it binds to the receptor RAGE on the cell membrane, acting as an alarm signal to influence a diverse processes in tumor initiation and progression by modulating downstream signaling pathways.

In terms of tumor proliferation, the HMGB1/RAGE axis promotes tumor cell growth through several signaling pathways, including NF-kB, K-Ras, MAPK, AKT, mTOR, STAT3, MEK, and ERK1/2. In terms of tumor apoptosis, the HMGB1/RAGE axis mediates tumor cell death through signaling pathways involving NF-kB, AKT, mTOR, and ERK1/2. It also enhances the expression of cell factors, including p65, p53, PARP, Bax, procaspase7, Bcl-2, Bcl-xl, and Cyt-C, further inhibiting tumor cell apoptosis. Inhibitors of the HMGB1/RAGE axis, such as EP, Quercetin, and Aloin, as well as gemcitabine (GEM), which suppresses HMGB1 and RAGE expression, can impact downstream signaling pathways.

Regarding tumor migration, invasion, and epithelial-mesenchymal transition (EMT), the HMGB1/RAGE axis upregulates the expression of cell factors like integrin α5β1, MMP1, MMP3, MMP9, and MMP10, through signaling pathways involving NF-kB, PI3K-AKT-mTOR, Snail, MAPK, TGF-β, STAT3, MEK-ERK1/2, thereby promoting tumor migration, invasion, and EMT. Inhibitors of the HMGB1/RAGE axis, such as microRNA-218, Glycyrrhizin, Verbascoside, Nifedipine, and EP, exert inhibitory effects. Moreover, the downregulation of small molecule RNAs like circRNA101368 and miRNA-200a can also inhibit the HMGB1/RAGE axis and subsequent signaling pathways.

In terms of tumor autophagy, the HMGB1/RAGE axis induces cellular autophagy through signaling pathways involving PI3K-AKT-MDR1, mTOR, NF-kB, MAPK, and p-ERK1/2. It upregulates the expression of cell factors like Bax, Beclin-1, LC3-II, Atg5, Beclin-1/VPS34 autophagosome complex, and p-Drp1, thereby promoting cellular autophagy. Compounds like Lucidone and GEM can inhibit the HMGB1/RAGE axis when used in combination, as can 4-AAQB. Regarding tumor angiogenesis, inhibiting the HMGB1/RAGE axis leads to a decrease in the expression of vascular endothelial growth factor (VEGF) and its receptor VEGFR2, thereby inhibiting tumor angiogenesis. EVO, an active compound found in Ampelopsis, acts as an anticancer agent by directly binding to HMGB1 and inhibiting the HMGB1/RAGE axis, ultimately inhibiting angiogenesis.

Considering the critical role of HMGB1/RAGE axis in tumor biology, HMGB1/RAGE may serve as a promising therapeutic target for cancers. Certain small molecule RNAs, such as microRNA-218 and lncRNA (TP73-AS1), can suppress the HMGB1/RAGE axis. Additionally, compounds like ethyl pyruvate (EP) and Epstein-Barr virus also exert inhibitory effects on this axis. This article comprehensively reviews the signaling pathways and molecular changes dependent on the HMGB1/RAGE axis, as well as various inhibitors of the HMGB1/RAGE axis, providing preliminary information for further research on the role of HMGB1/RAGE in tumors, as well as the development of targeted therapies and molecular inhibitors in clinical settings.

## Author contributions

AF: Writing – original draft. MG: Writing – review & editing. XT: Writing – review & editing. MJ: Writing – review & editing. CW: Writing – review & editing. YW: Writing – review & editing. QG: Writing – review & editing. JZ: Writing – review & editing.
